# The correlation between *Trichomonas vaginalis* infection and reproductive system cancer: a systematic review and meta-analysis

**DOI:** 10.1186/s13027-023-00490-2

**Published:** 2023-03-02

**Authors:** Zhenchao Zhang, Dongxian Li, Yuhua Li, Rui Zhang, Xianghuan Xie, Yi Yao, Linfei Zhao, Xiaowei Tian, Zhenke Yang, Shuai Wang, Xuejing Yue, Xuefang Mei

**Affiliations:** 1 Department of Pathogenic Biology, School of Basic Medical Sciences, Xinxiang Medical University, Xinxiang, Henan 453003 People’s Republic of China; 2 Xinxiang Key Laboratory of Pathogenic Biology, School of Basic Medical Sciences, Xinxiang Medical University, Xinxiang, Henan 453003 People’s Republic of China

**Keywords:** *Trichomonas vaginalis*, Infection, Cancer, Correlation, Systematic review

## Abstract

**Background:**

*Trichomonas vaginalis* (*T. vaginalis*) is a microaerophilic protozoan parasite which is responsible for trichomoniasis, the most common non-viral sexually transmitted infection in the world. The infection greatly damages the reproductive system. However, whether *T. vaginalis* infection can cause reproductive system cancer remains controversial.

**Methods:**

This study systematically searched PubMed, EMBASE, Ovid and Google scholar, and 144 relevant articles were retrieved and classified into three categories: epidemiological investigations (68), reviews (30) and research articles (46). These three types of articles were verified according to their respective inclusion and exclusion criteria. Stata 16 was used to conduct a meta-analysis on the articles of epidemiological investigations for analysing the correlation between *T. vaginalis* infection and reproductive system cancer.

**Results:**

The result of meta-analysis indicated that the rate of *T. vaginalis* infection in the cancer group was significantly higher than that in the non-cancer group (OR = 1.87, 95% CI 1.29–2.71, I^2^ = 52%). Moreover, the cancer rate of the population infected with *T. vaginalis* was significantly higher than that of the population without *T. vaginalis* infection (OR = 2.77, 95% CI 2.37–3.25, *I*^2^ = 31%). The review articles and most research articles stated that the infection of *T. vaginalis* could lead to cancer and the pathogenic mechanisms were as follows: *T. vaginalis* promoting inflammatory response, *T. vaginalis* infection changing the internal environment around parasitic sites and signal transduction pathway, the metabolites secreted by *T. vaginalis* inducing carcinogenesis and *T. vaginalis* increasing other pathogenic microbial infection to promote the occurrence of cancer.

**Conclusions:**

Our study confirmed that there was a correlation between the infection of *T. vaginalis* and reproductive system cancer, and provided some possible research directions for clarifying the carcinogenic mechanisms caused by *T. vaginalis* infection.

## Background

Trichomoniasis is the most common non-viral sexually transmitted infection (STI) in human beings which is responsible for a range of symptoms such as increasing vaginal secretion, pruritus and irritation of perineum in female [1]. According to the survey of the World Health Organization (WHO), at least 370 million people worldwide suffered from trichomoniasis. The global prevalence of trichomoniasis was 5.3% in females and 0.6% in males with a growing trend [2]. *Trichomonas vaginalis* (*T. vaginalis*) infection prevalence was 1.8% and 0.5% among females and males in the U.S [3]. In France, the total infection rates of *T. vaginalis* was 1.7% [4]. In Africa, the prevalence was as higher as 29% in Natal and 7.1% in Tanzania [5, 6]. In China, the prevalence was varied in different regions, for instance, 13.9% in Zhengzhou, 1.6% in Xinxiang [7] and 0.7% in Sichuan [8].

The infection of *T. vaginalis* can cause female vaginitis, cervicitis and adverse birth outcomes [9, 10]. Most of male infected with *T. vaginalis* are asymptomatic, but the infection can also cause urethritis or prostatitis and even lead to infertility [11]. Moreover, *T. vaginalis* usually increases the risk of other pathogens infection [12], for example HIV, which greatly threaten public health [13]. Some studies indicated that *T. vaginalis* infection might be a risk factor for cervical cancer and prostate cancer [14, 15].

As is known to all, the main pathogenic pathway of *T. vaginalis* is to cause the inflammatory reaction of parasitic sites, and the repeated inflammation may induce carcinogenesis. It was reported that *T. vaginalis* infection could lead to cervical precancerous lesions and neoplastic lesions [16]*.* The risk of cervical cancer increased about twice in the presence of *T. vaginalis* [17], and *T. vaginalis* infection could promote prostate cancer by damaging prostate epithelial cells [18]. Moreover, *T. vaginalis* infection was associated with hrHPV, the causative agent in most cervical cancers [13, 16].

Although many published articles have shown that *T. vaginalis* infection can increase the risk of cervical cancer or prostate cancer, the correlation between *T. vaginalis* infection and reproductive system cancer and whether *T. vaginalis* can cause reproductive system cancer remain unclear. Therefore, in this study, we analyzed the correlation between *T. vaginalis* infection and reproductive system cancer through meta-analysis of relevant epidemiological data. In addition, we summarized the potential pathogenic mechanism of cancer caused by *T. vaginalis* infection through consulting the relevant review and research articles. The results of the study provided a direction for exploring the pathogenic mechanism of *T. vaginalis* leading to cancer.

## Methods

### Search strategy and classification

The literature retrieval databases mainly included PubMed database, EMBASE, Ovid MEDLINE medical literature library, Web of Science, Science Direct and Google Scholar, and the literature search time was up to December 2021. The keywords searched by MeSH and commonly used for literature retrieval were used alone or in combination: “*Trichomonas vaginalis*”, “Trichomonas infections”, “Trichomoniasis”, “Trichomonas vaginitis”, “Neoplasms”, “cancer”, “neoplasia”, “carcinoma in situ”, “canceration” and “tumour” with “OR” and / or “AND” operators. The systematic search of literature was performed by two independent researchers. The retrieved articles were manually checked the title, abstract and full-text by two independent researchers, and the irrelevant articles and duplicate were removed. The retained articles were categorized as epidemiological investigations, reviews, and research articles. This systematic review with meta-analysis was registered in the International Prospective Register of Systematic Reviews (PROSPERO, https://www.crd.york.ac.uk/prospero) and a registration ID was assigned (CRD42022340263).

### Inclusion criteria and exclusion criteria

#### Inclusion criteria for epidemiological investigations

1. The article contains statistical data on cancer in people infected with *T. vaginalis* and without *T. vaginalis.* 2. The article contains statistical data on *T. vaginalis* infection in cancer patients and noncancer patients. 3. In the articles, the data of *T. vaginalis* infection in cancer patients and noncancer patients are compared. 4. All articles have clear data sources. 5. For the literature with repeated relevant data, the latest and most comprehensive article is selected.

#### Exclusion criteria for epidemiological investigations

1. The effect of *T. vaginalis* on cancer is not mentioned in the article, and the relevant data is incomplete. 2. The article only contains data on cancer in people infected with *T. vaginalis* with no corresponding data from people without *T. vaginalis* infection. 3. The article only contains data on *T. vaginalis* infection in cancer patients without corresponding data from noncancer patients. 4. In the articles, the sample size is too small.

#### Inclusion criterion for review articles

1. The article concerns the correlation between *T. vaginalis* infection and cancer. 2. The article has a clear conclusion.

#### Exclusion criterion for review articles

1. The article does not concern the correlation between *T. vaginalis* infection and cancer. 2. The conclusion about the correlation between *T. vaginalis* infection and cancer is ambiguous.

#### Inclusion criterion for research articles

There is a clear carcinogenic mechanism caused by *T. vaginalis* infection in the article.

#### Exclusion criterion for research articles

The relevant mechanism of cancer caused by *T. vaginalis* infection was not written in the article.

### Quality assessment and data analysis for epidemiological investigations

Newcastle–Ottawa scale (NOS) was used to evaluate the quality of the epidemiological investigations with a total score of 9 points, and the articles with a score ≥ 6 points were included in statistics. NOS considers the following items comprising selection criteria (0–4 points), subject comparability (0–2 points) and exposure (0–3 points). The selected epidemiological investigations were analyzed by Stata 16 software, and the forest plot was drawn. I^2^ test was used for heterogeneity analysis. If *P* < 0.05 or I^2^ ≥ 50%, it was considered that there was heterogeneity among the research, and the origin of heterogeneity should be further analyzed by the random-effect model and Galbraith plot. The funnel chart was used to test the publication bias of the included articles, and the Begg and egger tests were further used to evaluate the publication bias. If there was publication bias, we excluded articles one by one to make a sensitivity test, and the total effect quantity was observed to judge the stability of the analysis.

## Results

**Fig. 1:**
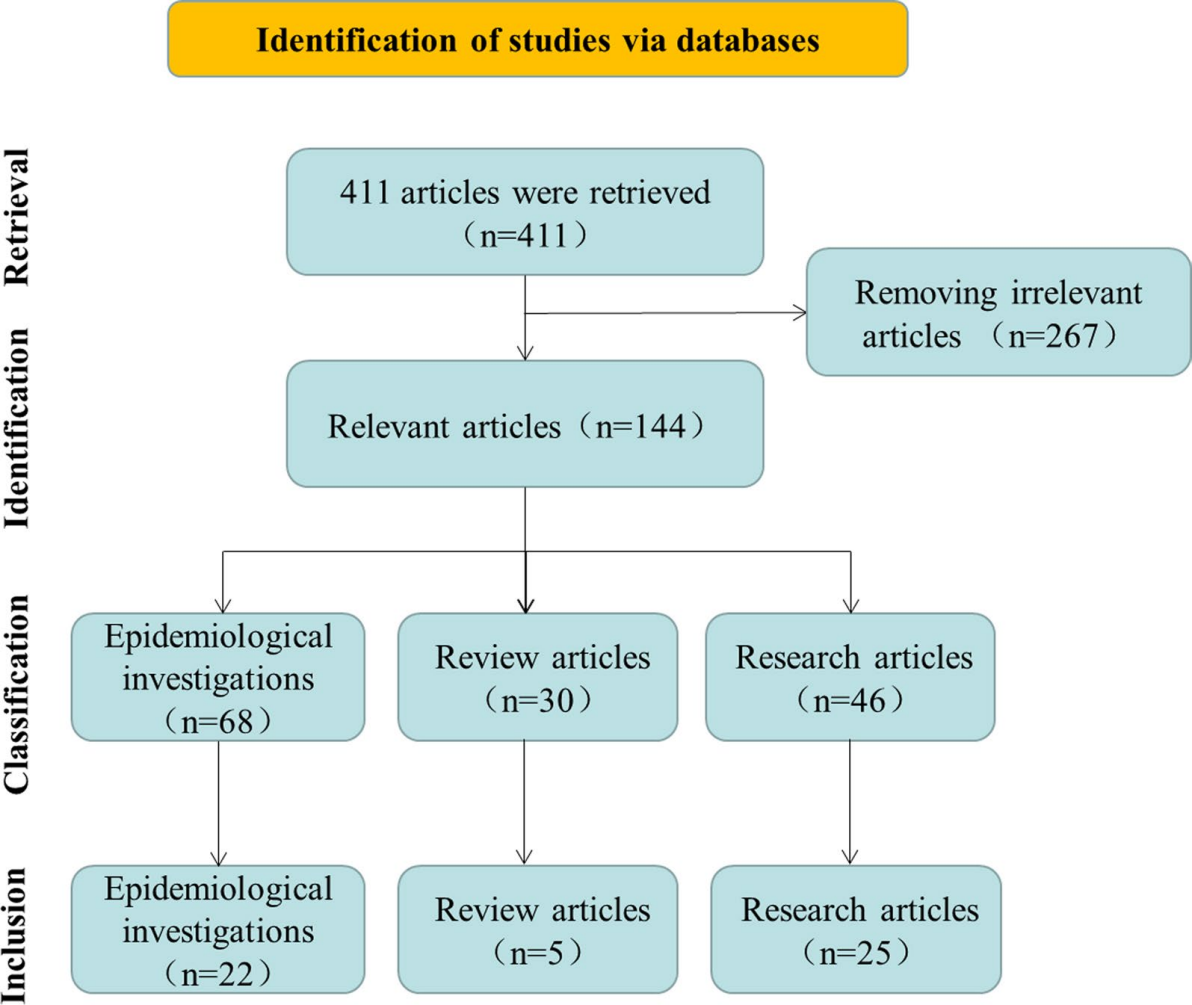
Flow diagram of articles retrieval, identification, classification and inclusion

In this study, a total of 411 articles were retrieved (Fig. 1), and the title, abstract and full-text of these articles were checked. After removing 267 irrelevant articles which did not mention both “*T. vaginalis*” and “Neoplasms”, the remaining 144 articles were classified into three categories, 68 epidemiological investigations, 30 review articles, and 46 research articles. In the 144 articles, 143 articles were about *T. vaginalis* and reproductive system cancer (cervical cancer, prostate cancer and vaginal cancer), and the other one was about *T. vaginalis* and anal canal carcinoma.

## Epidemiological investigations

### Literature inclusion

The 68 epidemiological selected articles were identified according to the NOS (NOS score ≥ 6), inclusion and exclusion criteria. Finally, 22 relevant articles were obtained. Fourteen articles (Table 1) were concerned the infection of *T. vaginalis* in reproductive system cancer patients. In these articles, we set the cancer patients as the experimental group and healthy people or noncancer patients as the control group. The other eight articles were concerned the reproductive system cancer incidence in *T. vaginalis* infected people. The people infected with *T. vaginalis* were set as the experimental group, and the people without *T. vaginalis* infection were set as the control group. The samples of these articles were sufficient for meta-analysis, which were deemed as high-quality articles based on the NOS quality assessment score (NOS score ≥ 6).

**Table 1:** The information of relevant epidemiological articles

Number	Title	First author	Year	Location	Total sample	Experimental group	Control group	Nos
1	An epidemiologic study of carcinoma in situ and squamous dysplasia of the uterine cervix	Thomas DB	1973	USA	626	49/324 (15.1%)	18/302 (6.0%)	8
2	The association of sexually transmitted diseases with cervical intraepithelial neoplasia: a case–control study	Guijon FB	1985	Canada	84	1/32 (3%)	1/52 (2%)	6
3	Vaginal microbial flora as a cofactor in the pathogenesis of uterine cervical intraepithelial neoplasia	Guijon F	1992	Canada	185	4/106 (3.8%)	4/79 (5.1%)	8
4	The association between sexually transmitted pathogens and cervical intra-epithelial neoplasia in a developing community	Kharsany AB	1993	Durban	48	11/28 (39%)	3/20 (15%)	6
5	*Trichomonas vaginalis* and cervical cancer. A prospective study in China	Zhang ZF	1995	China	16,797	7/421 (1.7%)	92/16376 (0.6%)	7
6	Atypical squamous cells of undetermined significance: Bethesda classification and association with Human *Papillomavirus*	Barcelos AC	2011	Brazil	170	4/140 (2.85%)	0/30 (0)	6
7	Prospective study of effect modification by Toll-like receptor 4 variation on the association between *Trichomonas vaginalis* serostatus and prostate cancer	Chen YC	2013	USA	1382	71/690 (10%)	61/692 (9%)	6
8	Bacterial vaginosis, aerobic vaginitis, vaginal inflammation and major Pap smear abnormalities Prevalence of human papillomavirus and co-existent sexually transmitted	Vieira P	2016	Portugal	1244	31/905 (3.4%)	11/339 (3.2%)	6
9	Prevalence of human papillomavirus, Chlamydia trachomatis, and Trichomonas vaginalis infections in Amazonian women with normal and abnormal cytology	Costa-Lira	2017	Brazil	180	0/47 (0)	24/133 (18%)	6
10	Correlation between Common Lower Genital Tract Microbes and High-Risk Human Papillomavirus Infection	Panpan Lv	2019	China	826	8/254 (3.1%)	1/572 (0.2%)	6
11	Prevalence of human *papillomavirus,* human immunodeficiency virus and other sexually transmitted infections among female sex workers in Togo: a national cross-sectional survey	Ferré VM	2019	Togo	310	9/102 (8.8%)	11/208 (5.3%)	6
12	Association between *Trichomonas vaginalis* infection and cervical lesions: a population-based, nested case–control study in Taiwan	Su RY	2020	Taiwan	270,015	12/54003 (0.02%)	18/216012 (0.008%)	6
13	*Trichomonas vaginalis* serostatus and prostate cancer risk in Egypt: a case–control study	Saleh NE	2021	Egypt	445	57/325 (17.5%)	10/120 (8.3%)	6
14	Prevalence of cervical HPV infection, sexually transmitted infections and associated antimicrobial resistance in women attending cervical cancer screening in Mali	Jary A	2021	Mali	144	7/90 (7.8%)	3/54 (5.6%)	7
15	Infection with *Trichomonas vaginalis* in a black population	Miller JM	1989	USA	3005	140/745 (18.8%)	169/2260 (7.5%)	6
16	Gynaecological infections as risk determinants of subsequent cervical neoplasia	Viikki M	2000	Finland	19,114	16/1544 (1.0%)	82/17600 (0.5%)	6
17	Association of *Chlamydia trachomatis* with persistence of high-risk types of human *papillomavirus* in a cohort of female adolescents	Samoff E	2005	USA	261	7/18 (39%)	83/234 (35.8%)	6
18	Results of longterm hospital based cytological screening in asymptomatic women	Misra JS	2006	India	20,417	54/671 (8.1%)	485/19746 (2.5%)	6
19	Human Papillomaviruses and genital co-infections in gynaecological Outpatients	Verteramo R	2008	Italy	857	3/10 (0.3%)	263/847 (1.2%)	7
20	Prevalence and risk factors for cervical neoplasia: a cervical cancer screening program in Beijing	Tao L	2014	China	748,105	9/5104 (1.8%)	504/697064 (0.07%)	6
21	Association between high risk human *papillomavirus* infection and co-infection with Candida spp. and *Trichomonas vaginalis* in women with cervical premalignant and malignant lesions	Ghosh I	2017	India	225	130/177 (73.4%)	27/48 (56.2%)	6
22	Trichomonas vaginalis as a risk factor for human *papillomavirus*: a study with women undergoing cervical cancer screening in a northeast region of Brazil	Belfort IKP	2021	Brazil	562	21/107 (20%)	27/455 (5.9%)	7

1–14 Experimental group: the number (incidence) of *T. vaginalis* infection among patients with cancer; Control group: the number (incidence) of *T. vaginalis* infection among people without cancer. 15–22 Experimental group: the number (incidence) of cancer among people with *T. vaginalis* infection; Control group: the number (incidence) of cancer among people without *T. vaginalis* infection

### Meta analysis of the epidemiological investigations

**Fig. 2:**
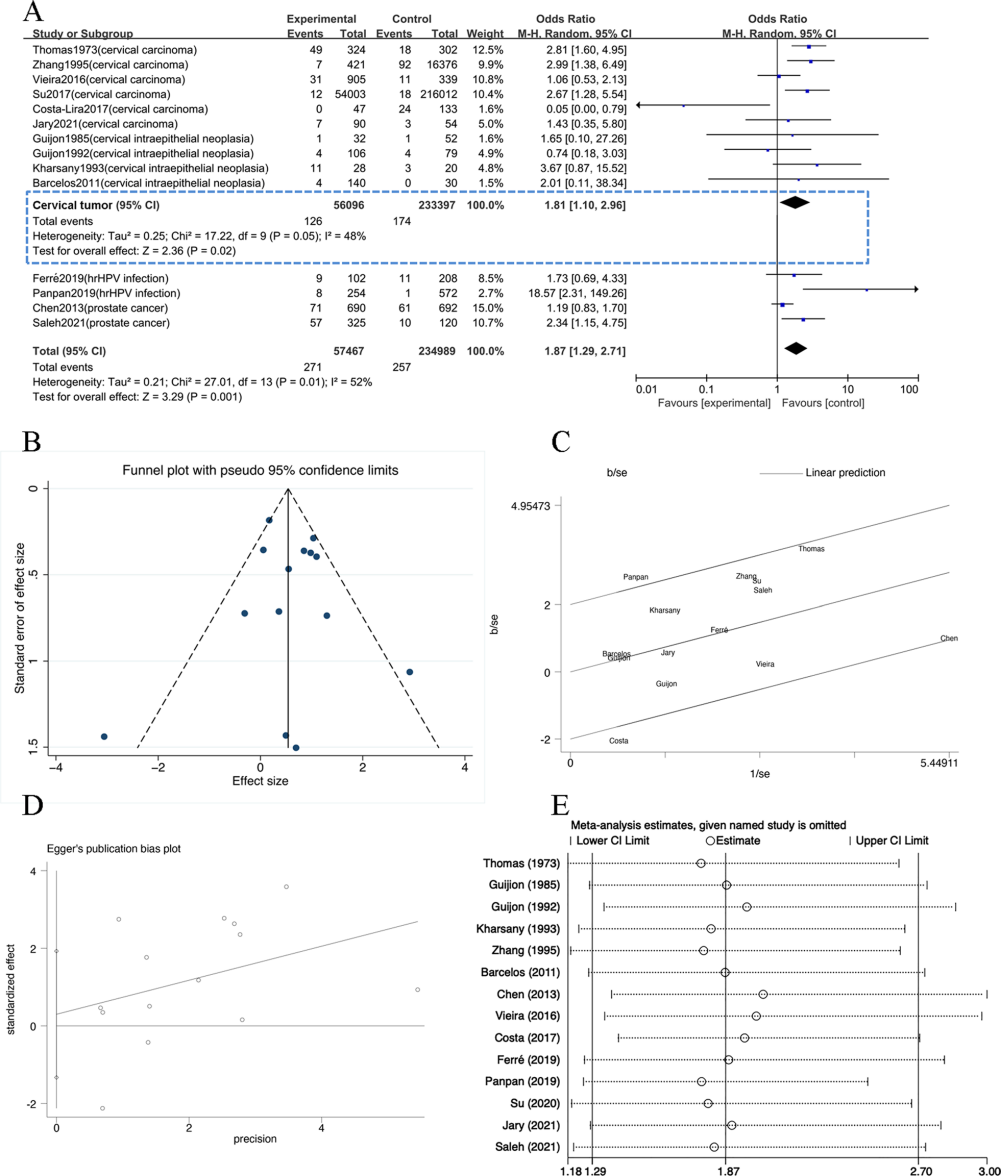
The articles concerned the incidence of *T. vaginalis* infection among patients with cancer. **A** Forest plot of the meta-analysis. The heterogeneity test yielded Chi^2^ = 27.01, *p* = 0.01, and I^2^ = 52%. The OR and 95% confidence interval were calculated as 1.87 and 1.29–2.71, respectively, and z and p values of the combined effect size were as 3.29 and less than 0.001. *T. vaginalis* infection among patients with cervical tumor: The heterogeneity test yielded Chi^2^ = 17.22, *p* = 0.05, and I^2^ = 48%. The OR and 95% confidence interval were calculated as 1.81 and 1.10–2.96, respectively, and z and p values of the combined effect size were as 2.36 and 0.02. **B** Funnel plot of the meta-analysis. **C** Galbraith plot. **D** Begg and Egger plot of the meta-analysis. *P* = 0.696 (*P* > 0.1) **E** Sensitivity analysis of the articles

The data of the 14 articles on *T. vaginalis* infection in cancer patients were analyzed by Stata 16 software. The results showed that 292,456 research subjects were included in the articles, including 57,467 in the experimental group (cancer patients) and 234,989 in the control group (healthy or non-cancer patients) [15, 19–31]. As shown in the forest plot (Fig. 2a), *P* = 0.01, I^2^ = 52% which indicated that there was heterogeneity among the research results (*P* < 0.1, I^2^ ≥ 50%). So random-effect model was used for further analysis, and Galbraith plot analysis was performed for the included articles (Fig. 2c). In the plot, the two articles, Panpan (2019) and Costa (2017), had influence on heterogeneity. The reason for the heterogeneity of Panpan (2019) was that the sample size was relatively small, while the reason for the heterogeneity of Costa (2017) was that the sample size of the experimental group and the control group was quite different.

Combining the data of these 14 articles, the pooled OR was 1.87 (95% CI 1.29–2.71). The results of the combined effect were Z = 3.29 and *P* = 0.001 which meant that there was a significant difference in *T. vaginalis* infection between the experimental group and the control group (*P* < 0.05). As shown in the funnel plot (Fig. 2b), the excellent distribution symmetry of the individual sample points indicated that there was no publication bias. Further, the Begg and Egger plot was used to test (Fig. 2d) the bias, and the result was found that *P* = 0.696 (*P* > 0.1) and showed that there was no publication bias in these data. The sensitivity analysis (Fig. 2e) found that the impact on the analysis was not significant after excluding each article, and the 95% confidence interval was still within 1.29–2.70, indicating that the analysis was relatively stable.

**Fig. 3:**
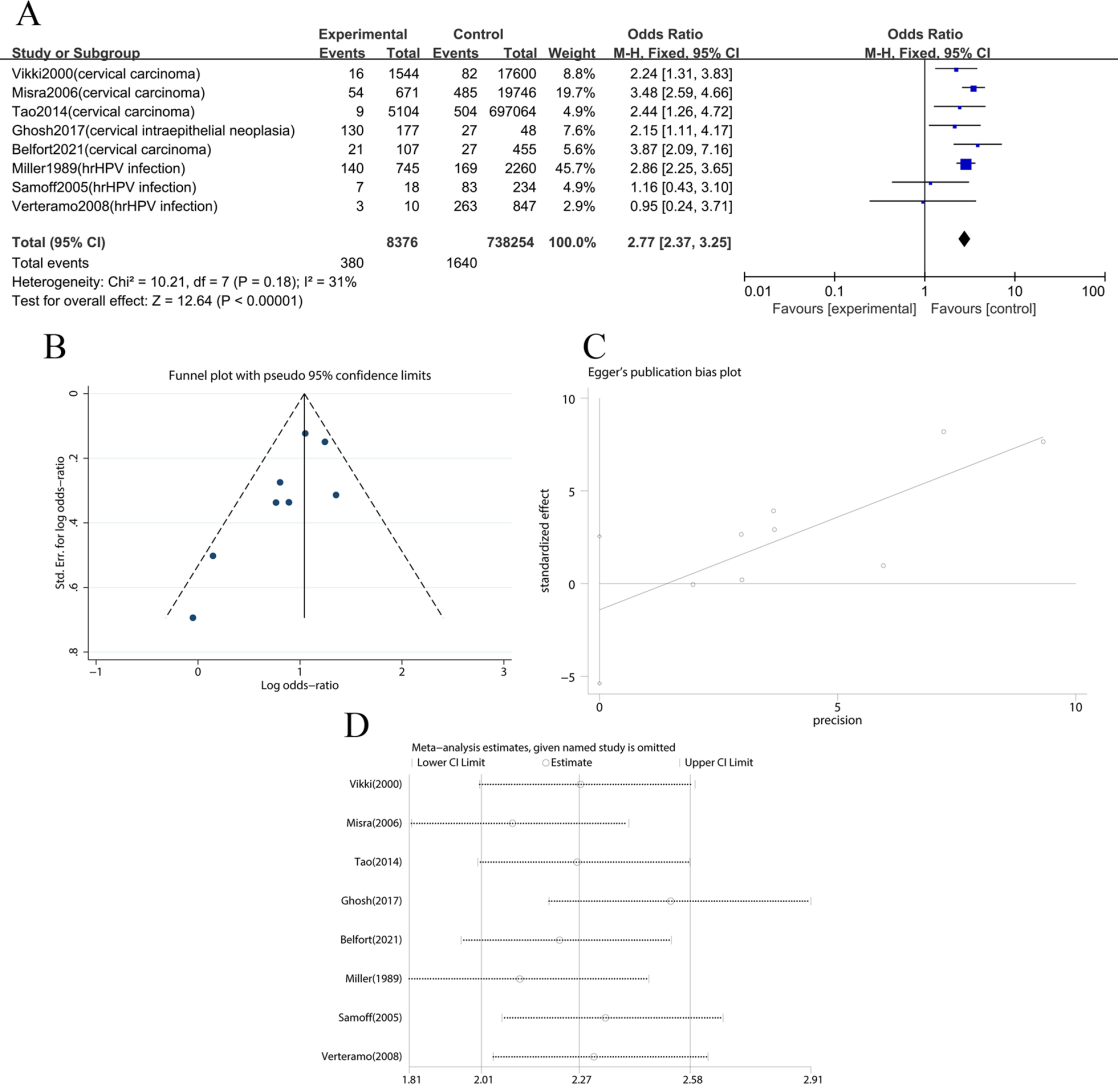
The articles concerned the incidence of cancer among people with *T. vaginalis* infection. **A** Forest plot of the meta-analysis. The heterogeneity test yielded Chi^2^ = 10.21, *p* = 0.18, and I^2^ = 31%. The OR and 95% confidence interval were calculated as 2.77 and 2.37–3.25, respectively, and z and p values of the combined effect size were as 12.64 and 0 (*P* < 0.05). **B** Funnel plot of the meta-analysis. **C** Begg and Egger plot of the meta-analysis. *P* = 0.051 (*P* < 0.1). **D** Sensitivity analysis of the articles

In addition, 8 articles on cancer among patients infected with *T. vaginalis* were screened for meta-analysis (Fig. 3a). The results showed that 746,630 subjects were included in the articles, including 8376 cases in the experimental group (with *T. vaginalis* infection) and 738,254 cases in the control group (without *T. vaginalis* infection) [32–38]. The pooled OR was 2.77 (95% Cl: 2.37–3.25). The results of the combined effect were Z = 12.64 and *P* = 0 (*P* < 0.05) which meant that there was a significant difference in cancer patients between the experimental group and the control group (*P* < 0.05). The results of I^2^ = 31%, *P* = 0.18 proved that the heterogeneity was small (*P* > 0.1). The funnel plot (Fig. 3b) were asymmetric, indicating that there might be publication bias. The Begg and Egger plot was used to test (Fig. 3c), *P* = 0.051 (*P* < 0.1) proved that there was publication bias. Because few retrieved articles were included, so it was necessary to add more articles for further study. The sensitivity analysis (Fig. 3d) indicated that the analysis was relatively stable (95%Cl: 2.01**–**2.58).

## Review articles

In this study, 30 reviews were selected and five articles were finally obtained after screening by inclusion and exclusion criteria. As shown in Table 2, we analyzed the conclusions of each article. These five articles suggested that *T. vaginalis* could lead to cervical cancer and prostate cancer [16, 17, 39–41]. The possible reasons were cell carcinogenesis caused by long-term inflammatory response [16, 17, 39, 40] and cancer caused by *T. vaginalis* increasing the infection of other pathogens (hrHPV, *Chlamydia*) [41].

**Table 2:** The information of review articles

Title	First author	Year	Conclusion	*T. vaginalis* causes cancer
Infection and cervical intraepithelial neoplasia	Boyle DC	1999	The risk of cervical neoplasia in the presence of *T. vaginalis* is about twice that in normal, which may be related to the production of nitrosamine	Yes
*Trichomonas vaginalis*: paradigm of a successful sexually transmitted organism	Rughooputh S	2005	*T. vaginalis* can be classified as one of the most important auxiliary factors in the pathogenesis of cervical cancer	Yes
Sexually transmitted infections and risk of prostate cancer: review of historical and emerging hypotheses	Sutcliffe S	2014	*T. vaginalis* promotes prostate cancer through an IgE mediated anti flagellar hormone inflammatory immune mechanism, while *T. vaginalis* may promote cancer by directly damaging or dissolving prostate epithelial cells	Yes
Association of Genital Infections Other Than Human Papillomavirus with Pre-Invasive and Invasive Cervical Neoplasia	Ghosh I	2016	*T. vaginalis* infection has a higher risk of cervical precancerous lesions and neoplastic lesions	Yes
The dawn of novel STI prevention methods: modelling potential unintended effects of changes in cervical cancer screening guidelines on *trichomoniasis*	Rönn MM	2018	Patients infected with hrHPV are more likely to be infected with *T. vaginalis* than those not infected with hrHPV	Yes

## Research articles

In this study, 48 research articles were selected and 25 articles were obtained according to the inclusion and exclusion criteria of research articles, of which 24 articles were related to *T. vaginalis* infection and reproductive system cancer, and the other articles involved *T. vaginalis* infection and anal canal carcinoma [18, 42–67]. As shown in Table 3, we listed the information of the articles and concluded the mechanism of cancer caused by *T. vaginalis* infection. Most of the articles showed that *T. vaginalis* infection could promote cancer procession but 3 articles hold the opposite view. As described in these articles, the main pathogenic mechanisms were as follows: (1) *T. vaginalis* promoted inflammatory reaction through various ways and leaded to cell carcinogenesis; (2) The metabolites secreted by *T. vaginalis* promoted the occurrence of cancer; (3) The infection of *T. vaginalis* affected the vivo environment and signal transduction pathway which was associated with cancer; (4) *T. vaginalis* increasing other pathogenic microbial infection (HPV) promoted the occurrence of cancer.When phagocytosis of *Candida* spp. by *T. vaginalis* occurs, *Candida* spp. are protected by *T. vaginalis* from the defences of the host and the inhibitory effects of antimycotic drugs used for treatment finally lead to anal canal carcinoma

**Table 3:** The information of research articles

Title	First author	Year	Conclusion	*T. vaginalis* causes cancer
Significance of variations in the size of *Trichomonas vagina*lis in patients with dysplasia, intrapithelial and invasive planocellular carcinoma of the uterine cervix	Mekki F	1979	Small forms of *T. vaginalis* are more pathogenic than large ones and might be one of the causative agents of the atypical transformation of the squamous epithelium of the uterine cervix	Y*es*
Enhancement versus tumor resistance induced by different levels of immunodepression in BALB/c mice with protozoan infections	Landolfo S	1979	In *T. vaginalis* infected mice, a slight and transient depression of both humoral and cellular immune reactivity induces an enhanced tumor growth	Y*es*
Gas chromatographic studies on propionic acid, butyric acid and valeric acid in culture fluid of *Trichomonas vagina*lis	Ishiguro T	1984	Propionic acid or iso-valeric acid produced by *T. vaginalis* had a promoter-like activity and/or promoter-enhancing effect, which is, at least in part, responsible for the promotion of cervical cancer or vaginal cancer	Yes
Pseudocyst forms of *Trichomonas vagina*lis *from cervical neoplasia*	Afzan MY	2012	*T. vaginalis* phenotypic variant forms of pseudocysts does exist and this phenotype with higher nuclear content and more rough and creased surface with higher numbers of deep micropores with larger numbers of chromatin masses, vacuoles, and hydrogenosomes play a role in exacerbating cervical cancer	Yes
Phenotypic 'variant' forms of Trichomonas vaginalis trophozoites from cervical neoplasia patients	Yusof AM	2012	*T. vaginalis* trophozoites in cervical neoplasia isolates showed more rough and creased surface with numerous deep micropores, and there was higher numbers of vacuoles and hydrogenosomes in these forms. These were virulent forms which could aggravate or exacerbate cervical neoplasia conditions	Yes
Light microscopic observation on phagocytosis of Candida spp. *blastospores by Trichomonas vagina*lis *in a patient with anal canal carcinoma*	Oz ZS	2012		Y*es*
Epitopes of the highly immunogenic *Trichomonas vaginalis α*-actin in are serodiagnostic targets for both women and men	Neace CJ	2013	There is a relationship between seropositivity for α-actinin truncated protein of *T. vaginalis* and prostate cancer	Y*es*
Association of *Trichomonas vagina*lis with its symbiont Mycoplasma hominis synergistically upregulates the in vitro proinflammatory response of human monocytes	Fiori PL	2013	The synergistic upregulation of the macrophage proinflammatory response might also affect some important clinical conditions associated with *T. vaginalis* infection, such as the increased risk of acquiring cervical cancer or HIV, which are thought to be affected by the inflammatory milieu during trichomoniasis	Yes
*Trichomonas vaginalis* homolog of macrophage migration inhibitory factor induces prostate cell growth, invasiveness, and inflammatory responses	Twu O	2014	Chronic *T. vaginalis* infections may result in TvMIF-driven inflammation and cell proliferation, thus triggering pathways that contribute to the promotion and progression of prostate cancer	Yes
*Trichomonas vaginalis:* a possible foe to prostate can*cer*	Zhu Z	2016	*T. vaginalis* inhibits the growth and development of prostate cancer	No
Signalling pathways associated with IL-6 production and epithelial-mesenchymal transition induction in prostate epithelial cells stimulated with *Trichomonas vagina*lis	Han IH	2016	Inflammatory conditions induced by *T. vaginalis* infections have been shown to promote epithelial-mesenchymal transition (EMT)	Yes
Proliferation of Prostate Stromal Cell Induced by Benign Prostatic Hyperplasia Epithelial Cell Stimulated With *Trichomonas vagina*lis via Crosstalk With Mast Cell	Kim JH	2016	The inflammatory response by benign prostatic hyperplasia epithelial cells stimulated with *T. vaginalis* induce the proliferation of prostate stromal cells via crosstalk with mast cells	Yes
Inflammatory Responses in a Benign Prostatic Hyperplasia Epithelial Cell Line (BPH-1) Infected with *Trichomonas vaginalis*	Kim SS	2016	The level of IL-6 in BPH-1 cells infected with *T. vaginalis* increased, and IL-6 is considered to promote the development of benign prostatic hyperplasia and prostate cancer	Yes
*Trichomonas Vaginalis* Inhibits *HeLa* Cell Growth Through Modulation of Critical Molecules for Cell Proliferation and Apoptosis	Zhu Z	2018	*T. vaginalis* culture supernatant inhibited the growth of HeLa cervical cancer cells by inhibiting cell proliferation and promoting apoptosis	No
Druggability of the guanosine/adenosine/cytidine nucleoside hydrolase from *Trichomonas vagina*lis	Alam R	2018	Individuals infected with *T. vaginalis* have a higher susceptibility to more serious conditions such as cervical and prostate cancer	Yes
Inflammatory mediators of prostate epithelial cells stimulated with *Trichomonas vaginalis* promote proliferative and invasive properties of prostate cancer cells	Han IH	2019	*T. vaginalis* infection may be one of the factors creating the supportive microenvironment to promote proliferation and invasiveness of PCa cells	Yes
Experimental rat prostatitis caused by *Trichomonas vagina*lis infection	Jang KS	2019	*T. vaginalis* has been detected in prostatic tissue of patients with prostatitis and reported to be associated with chronic prostatitis and benign prostatic hyperplasia as well as prostate cancer	Y*es*
IL-6 produced by prostate epithelial cells stimulated with *Trichomonas vaginalis* promotes proliferation of prostate cancer cells by inducing M2 polarization of THP-1-derived macrophages	Han IH	2020	When *T. vaginalis* infection causes inflammation, prostate epithelial cells produce IL-6, macrophages polarize into M2 type, and M2 macrophages promote the proliferation and migration of cancer cells	Yes
*Gardnerella vaginalis* and *Trichomonas vaginalis* infections and the risk of persistence or progression of low-grade cervical intraepithelial neoplasia	Raffone A	2020	*T. vaginalis* infection alone does not significantly affect the risk of persistence or progression of such lesions, while it may greatly increase the risk of progression when associated with *G. vaginalis* infection	Yes
Polarization of M2 Macrophages by Interaction between Prostate Cancer Cells Treated with *Trichomonas vaginalis* and Adipocytes	Chung HY	2020	Interaction between inflamed PCa treated with *T. vaginalis* and adipocytes causes M2 macrophage polarization, so contributing to the progression of PCa	Yes
The Role of Purinergic Signaling in *Trichomonas vagina*lis Infection	Ferla M	2020	*T. vaginalis* infect the prostate and make prostate epithelial cells express P2X1, P2X2 and P2X7 receptors, affecting the purinergic signaling of host, which may be related to prostate cancer	Yes
Inflammation driven tumor-like signaling in prostatic epithelial cells by sexually transmitted *Trichomonas vagina*lis	Kushwaha *B*	2020	The initiation of inflammation driven tumor-like cell signaling in parasite-infected human prostatic epithelial cells is apparent, with the prostate tumor (DU145) cells being more sensitive to *T. vaginalis* than normal (RWPE-1) prostatic cells	Yes
Investigation of viral etiology in potentially malignant disorders and oral squamous cell carcinomas in non-smoking, non-drinking patients	Pérot P	2020	*T. vaginalis* can induce the production of a large number of different proinflammatory cytokines, which is associated with a high risk of high-grade or metastatic prostate cancer	Yes
Molecular Examination of *Trichomonas vaginalis* Infection and Risk of Prostate Cancer in the Biopsy of Patients with Different Prostate Lesions	Kamarkhani Z	2021	*T. vaginalis* may have no pathogenic effect on different prostate lesions	No
Signaling Role of Adipocyte Leptin in Prostate Cell Proliferation Induced by *Trichomonas vaginalis*	Kim JH	2021	*T. vaginalis* contributes to prostate enlargement in BPH via adipocyte leptin released as a result of inflammation of the prostate	Yes

## Discussion

In recent years, there are increasing number of studies on *T. vaginalis*, and many studies indicated that *T. vaginalis* was closely related to reproductive system cancer. In this study, we searched for 14 articles on incidence of *T. vaginalis* infection in cancer patients. By meta-analysis, the combined effect size of the forest plot was determined to Z = 3.29, *P* = 0.001, which was statistically significant. However, the analysis result of forest plot (*P* = 0.01, I^2^ = 52%) indicated a moderate degree of heterogeneity in these articles. Galbraith plot indicated that there was heterogeneity in Panpan (2019) and Costa (2017), but sensitivity analysis showed that the removal of the 2 articles has little impact on the results, and the data was stable. These results signified that the infection rate of *T. vaginalis* in cancer patients was significantly higher than that in healthy group or non cancer group, which indicated there was a correlation between *T. vaginalis* infection and cancer. By analyzing funnel plot, Begg and Egger plot, we found that there was no publication bias in our analysis. However, the heterogeneity still affected the quality of the analysis. Although the result indicated that *T. vaginalis* infection was related to cancer, more epidemiological data should be further excavated.

In addition, there were eight epidemiological investigations on incidence of cancer in people with *T. vaginalis* infection. In the study, the people infected with *T. vaginalis* were set as the experimental group, and the people without *T. vaginalis* infection were set as the control group. By comparing the prevalence of cancer between the two groups, the results indicated that the group infected with *T. vaginalis* had a high proportion of cancer. There was no heterogeneity in the data (I^2^ = 31%, *P* = 0.18). However, the Begg and egger analysis of the data (*P* = 0.051, *P* < 0.1) showed publication bias in the result. The reason for this might be related to the small number of such articles in this study. Therefore, the epidemiological investigations on the prevalence of cancer in the people infected with *T. vaginalis* need further research and more relevant articles need to be included to consolidate the results.

Several review articles on *T. vaginalis* and cancer were selected in this study. By consulting these articles, we found that *T. vaginalis* plays a positive role in the occurrence and evolution of cancer. *T. vaginalis* infection could directly or indirectly lead to cancer. It was found that *T. vaginalis* infection in women mainly caused inflammation of reproductive tract and the release of proinflammatory factors, changed in vaginal environment and promoted pathogenic microbial infections (HPV) [16, 41] which led to cervical cancer. The main factors leading to prostate cancer in men infected with *T. vaginalis* were the inflammatory response induced by *T. vaginalis* or its cytotoxic effect. The infection of *T. vaginalis* could induce the production of a large number of different proinflammatory cytokines [40].

From the research articles, we found several potential mechanisms of cancer induced by *T. vaginalis* infection.

The infection of *T. vaginalis* can promote the inflammatory response and lead to cell carcinogenesis in a variety of ways. Chronic *T. vaginalis* infection leads to the production of macrophage migration factor and macrophage polarization to M2 type, driving inflammation and abnormal cell proliferation to promote the progress of cervical and prostate cancer [60, 62]. The inflammatory response by BPH (benign prostatic hyperplasia) epithelial cells stimulated with *T. vaginalis* induce the proliferation of prostate stromal cells via crosstalk with mast cells [55, 67]. Chronic *T. vaginalis* infections result in TvMIF (*T. vaginalis* macrophage migration inhibitory factor)-driven inflammation and cell proliferation, thus triggering pathways that contribute to progression of prostate cancer [51]. The initiation of inflammation driven tumor-like cell signaling in parasite-infected human prostatic epithelial cells and the prostate tumor cells are more sensitive to *T. vaginalis* than normal prostatic cells [63]. The level of IL-6 in BPH-1 cells infected with *T. vaginalis* is increased, and IL-6 is considered to be a factor promoting the development of benign prostatic hyperplasia and prostate cancer [54, 66].

During the metabolism of *T. vaginalis*, the contents of propionic acid and iso-valeric acid increase [44]. Both of them had a promoter-like activity and/or promoter-enhancing effect in vitro studies on viral oncology, which could promote the occurrence of cervical cancer or vaginal cancer. Moreover, *T. vaginalis* infect the prostate cells and lead prostate epithelial cells to express P2X1, P2X2 and P2X7 receptors, affecting the purinergic signaling of host, which is related to prostate cancer [18, 47]. *T. vaginalis* count and growth rates were significantly higher in trophozoites from CN (cervical neoplasia) and cervical neoplasia proliferates, and the trophozoite surface of CN isolate was creased and rough implying that these were virulent forms which could aggravate cervical neoplasia conditions [47]. There is a relationship between seropositivity for ACT-P2 (truncated protein of trichomonad α-actinin) of *T. vaginalis* and prostate cancer [49]. *T. vaginalis* may promote cancer by directly damaging or dissolving prostate epithelial cells [52].

*T. vaginalis* infection associated with other pathogen infection increase the risk of cancer. The presence of *Mycoplasma hominis* (*M. hominis*) in *T. vaginalis* play a key role in inflammation. The synergistic upregulation of the macrophage proinflammatory response also increase the risk of acquiring cervical cancer [50]. When phagocytosis of Candida spp by *T. vaginalis* occurs, Candida spp are protected by *T. vaginalis* from the defences of the host and the inhibitory effects of antimycotic drugs used for treatment finally lead to anal canal carcinoma [48]. *T. vaginalis* infection associated with *Gardnerella vaginalis* infection might increase the progression of low-grade cervical intraepithelial neoplasia [61]. Morever, some published articles indicated that *T. vaginalis* significantly increased the infection of hrHPV [41]. However, which mechanism plays a major role in cancer induced by *T. vaginalis* infection needs to be further studied.

## Conclusions

Through meta-analysis of relevant epidemiological data, we found that there was a correlation between *T. vaginalis* infection and reproductive system cancer. By consulting the relevant review and research articles, we discovered that *T. vaginalis* infection could lead to cervical cancer or prostate cancer, and the articles showed that the main potential carcinogenic mechanisms involved inflammatory reaction change the environment around the parasitic site and signal transduction pathway, and aggravate the infection of other carcinogenic pathogenic microorganisms. Our study provides a foundation for further investigation to the mechanism of cancer caused by *T. vaginalis* in the future.

## Data Availability

All data generated or analyzed during this study are included in this article and additional information files.

## Abbreviations

*T. vaginalis*: *Trichomonas vaginalis*
STI: Sexually transmitted infection
BPH: Benign prostatic hyperplasia
CN: Cervical neoplasia
ACT-P2: Truncated protein of trichomonad α-actinin
TvMIF: *T. vaginalis* Macrophage migration inhibitory factor
GV: *Gardnerella vaginalis*
hrHPV: High risk Human papilloma virus
*M**.** hominis*: *Mycoplasma hominis*
OR: Odds ratio
NOS: Newcastle–Ottawa scale
